# Optimizing an Enzymatic Extraction Method for the Flavonoids in Moringa (*Moringa oleifera* Lam.) Leaves Based on Experimental Designs Methodologies

**DOI:** 10.3390/antiox12020369

**Published:** 2023-02-03

**Authors:** Curro Polo-Castellano, Rosa María Mateos, Francisco Visiedo, Miguel Palma, Gerardo F. Barbero, Marta Ferreiro-González

**Affiliations:** 1 Department of Analytical Chemistry, Faculty of Sciences, Agrifood Campus of International Excellence (ceiA3), Wine and Food Research Institute (IVAGRO), University of Cadiz, 11510 Puerto Real, Spain; 2 Research Unit, Biomedical Research and Innovation Institute of Cadiz (INiBICA), Puerta del Mar University Hospital, 11009 Cadiz, Spain; 3 Area of Biochemistry and Molecular Biology, Department of Biomedicine, Biotechnology and Public Health, University of Cadiz, 11519 Cadiz, Spain

**Keywords:** *Moringa oleifera* Lam., extraction method, antioxidant power, phenolic compounds, flavonoids, DPPH

## Abstract

*Moringa oleifera* Lam. is known to have significant antioxidant properties. Because of this, the development of an optimal extraction method is crucial to obtain pharmacological products based on the bioactive compounds produced by this tree. Through a Plackett–Burman and a Box–Behnken design, enzymatic extraction conditions (temperature, agitation, solvent pH and composition, sample-to-solvent ratio, enzyme-to-sample ratio and extraction time) have been optimized using normalized areas (UA/g) as response variable and relative mass (mg/g) as quantification variable. Extractions were performed in an incubator, where all the extraction conditions could be digitally controlled. Thus, 58.9 °C, 50 rpm, 4.0 pH, 32.5% EtOH, 0.2 g sample in 15 mL solvent and 106 U/g were established as the optimal extraction conditions for the extraction with a mix of pectinases coming from *Aspergillus niger*. Under these optimal conditions, two-minute extractions were performed and evaluated through a single factor design. The enzymatic extraction method demonstrated its suitability to produce extracts with good antioxidant power (antioxidant activity 4.664 ± 0.059 mg trolox equivalent/g sample and total phenolic compounds 6.245 ± 0.101 mg gallic acid equivalent/g sample). The method was also confirmed to have good repeatability (1.39%) and intermediate precision (2.37%) levels.

## 1. Introduction

Natural products (NPs) are natural compounds or substances produced by a living organism, which are found in nature. Owing to their structural diversity, they represent a relevant source of lead compounds [1] suitable for the development of new drugs. Many NPs present structural similarities with certain endogenous compounds [2]. These similarities confer on them the ability to interact with certain body receptors and trigger their intrinsic activity, which results in a therapeutic effect on living organisms. These natural products can be isolated from different sources, where marine [3], animal [4], microbial [5] and vegetal [6,7] are some of the most representative ones, while the latter is the most frequently investigated nowadays.

The most common method to search for bioactive compounds consists of screening different sources that have previously demonstrated to have a considerable therapeutic potential [8]. As mentioned above, vegetal sources have been widely investigated, which has led to the discovery of an extensive variety of compounds with pharmacological activity and potential against certain health disorders.

*Moringa oleifera* Lam. is a native tree from north India [9]. It is commonly known as the miracle tree [10], and it is traditionally known to have a substantial medicinal potential. Its leaves, pods and flowers have been extensively used for the treatment of certain metabolic disorders such as hypercholesterolemia [10] or diabetes [11]. Its bioactivity is fundamentally attributed to the presence of an enormous variety of NPs in its leaves, flowers and roots (proteins, vitamins, glucosinolates, alkaloids, flavonoids, phenolic compounds…) [9,10,12] that confer this plant with its therapeutic potential as an antioxidant [13], anti-inflammatory [10,14], anti-hyperlypidemic [10] and hypoglycemic [11] agent. All the abovementioned properties make *M. oleifera* a good candidate to have its different parts screened in search of NPs that may have a therapeutical application regarding the treatment of a series of health disorders.

Phenolic compounds are common vegetal bioactive compounds, mainly known because of their antioxidant properties that make them suitable for the prevention of certain health issues related to oxidative processes, such as cancer or cardiovascular diseases [15]. In fact, *M. oleifera* is known to produce a large diversity of phenolic compounds (myricetin, quercetin and kaempferol derivatives, gallic and chlorogenic acid…) [16,17] that can be classified as flavonoid (anthocyanins, flavones, flavanones, flavanols, lignans…) [18] or non-flavonoid compounds (phenolic acids and non-carboxylic phenols) [19]. Flavonoids have been largely confirmed to act as antioxidant agents, as they can donate electrons to free radicals, which block the deleterious chain reactions involved in some degenerative processes, such as inflammation, diabetes or certain cardiovascular diseases [20,21]. Therefore, flavonoid-rich vegetal products, such as green tea [22] or wine [21], present potent antioxidant properties. Given the abundance of flavonoids in moringa plants, they are to be considered a great source of antioxidant compounds, where quercetin, kaempferol and their derivatives (quercetin 3-*O*-rhamnoside, kaempferol glucosides…) are the most representative ones [16,23,24].

Previous studies have been performed in this field, obtaining moringa extracts with phenolic contents of 20.16 mg gallic acid equivalents per gram of sample (GAE/g) [25] or with a total phenolic content between 23.22 ± 1.12 and 43.96 ± 1.90 mg/g [26] by using non-conventional extraction techniques, such as microwave-assisted extraction (MAE) or optimized ultrasound-assisted extraction (UAE).

The composition of the moringa extracts depends to a large extent on the plant tissue used as a sample, the extraction method and the specific extraction conditions [27]. For that reason, before proceeding to any massive extractions, the design and optimization of an appropriate extraction method as well as the evaluation of the composition of the different sample sources (roots, flowers, leaves…) must be carried out. In this regard, enzymatic extraction (EE) has proven to be one of the most effective methods for the extraction of bioactive compounds, as it employs enzymes that catalytically break the plant cell walls and membranes, thus releasing all the compounds that are present in the cells into the solvent [28]. When compared against other extraction techniques, such as ultrasound assisted extraction (UAE) or microwave assisted extraction (MAE), it can be seen that EE presents several advantages that support its position as the most suitable extraction method for this purpose. These advantages include scalability, high extraction yields [29] and the fact that it can be implemented under mild conditions, which contributes to preserving the structural integrity of the bioactive compounds of interest [28]. Furthermore, EE can be complemented with other techniques, such as UAE [30]. Cellulases and pectinases are some of the enzymes most frequently used in EE, as cellulose and pectin are the most representative components in vegetal cell walls [31,32]. Apart from the type and amount of enzyme employed, other factors, such as the pH of the solvent and its composition, the temperature, agitation and the sample-to-solvent ratio as well as the extraction time [33], are also variables to be taken into account for an optimal design of the extraction procedure.

Considering all of the factors mentioned above, the present study intends to develop an efficient EE method for obtaining the bioactive extracts that can be found in *M. oleifera* and to demonstrate the antioxidant power of such extracts based on a number of chemical assays. The novelty of this study lies in the fact that a higher number of factors than usual is initially considered for the statistical design, which combines a screening Plackett–Burman design for the determination of the factors with a higher significance and a surface-response Box–Behnken design for their optimization [34,35]. It also intends to employ a greener technique (EE) than those used for conventional extraction methods (UAE, MAE) as it employs lower temperatures, lower amounts of organic solvents and requires less extraction times for the obtention of the extracts.

## 2. Materials and Methods

### 2.1. Plant Material

Dried leaves of *M. oleifera* were provided by the company Connatur (36.334872, −6.111330; Conil, Spain), which specialises in the cultivation and commercialisation of moringa. The dried leaves were ground in a conventional mill and sieved to a pore size of 200 mesh.

### 2.2. Solvents and Reagents

The extraction solvents consisted of variable percentages of absolute ethanol EssentQ^®^ (Scharlab, Barcelona, Spain) diluted in different citrate/phosphate buffers at pH 4.0, 5.0 and 6.0 obtained by solving calcium citrate (Panreac S.L.U., Castellar del Vallés, Spain) and sodium dihydrogenphosphate (Panreac S.L.U., Castellar del Vallés, Spain) in Milli-Q water obtained by filtration through a MilliPore system (Bedford, MA, USA). The enzyme employed for the extraction was a pectinase obtained from *Aspergillus niger* (Merck KgaA, Darmstadt, Germany).

For the determination of the antioxidant capacity of the extract, 2,2-diphenyl-1-picrylhydrazyl (DPPH) and Trolox (Merck KgaA, Darmstadt, Germany) were used.

In order to quantify the amount of phenolic compounds present in the extract, gallic acid, Folin–Ciocalteu reagent and sodium carbonate (Merck KgaA, Darmstadt, Germany) were employed.

Two solvents were employed to separate the constituents of the extracts as follows: HPLC-grade acetonitrile (Panreac S.L.U, Castellar del Vallés, Spain) and Milli-Q water, both acidified using 2% glacial acetic acid (Panreac S.L.U, Castellar del Vallés, Spain). Quercetin 3-*O*-glucoside (Q3GLU) (Merck KgaA, Darmstadt, Germany), were used as standard for the quantification.

### 2.3. Enzymatic Extraction

#### 2.3.1. Extraction Equipment

The equipment used for the extraction was a Nahita LNB001 (Auxilab S.L., Navarra, Spain) incubator fitted with a touch screen that allows digitally setting up agitation, temperature and time values.

#### 2.3.2. Extraction Procedure

Several factors were considered for the optimization of the extraction method as follows: solvent composition (0−40% ethanol in water) and pH (4.0−6.0), agitation (50−200 rpm), temperature (40−60 °C), sample-to-solvent ratio (0.1−0.2 g in 15 mL solvent), enzyme-to-sample ratio (100−1000 U/g) and extraction time (10−60 min). Levels for each factor were stablished according to previous studies of our research group [36]. The enzyme amount was determined according to other studies of enzymatic extraction with pectinases [21].

The samples for the extractions were weighted and mixed with the corresponding solvent for each experiment. Then, the enzyme was added, and the extraction conditions were set up to proceed with the extraction. Once the extraction had been completed, the resulting extracts were centrifuged twice (1702× *g*, 5 min). The supernatant was collected, made up to 25 mL and kept in Falcon tubes until further analysis.

### 2.4. Phenolic Compounds Quantification

The compounds were separated by means of an ACQUITY UPLC^®^ H-Class System (Waters Corporation, Milford, MA, USA). This equipment consists of a quaternary elution system (Quaternary Solvent Manager) coupled to a photodiode array detector (PAD eλ Detector, Waters Corporation, Milford, MA, USA). The column used for the separation was an ACQUITY UPLC^®^ BEH C18 (1.7 μm, 2.1 mm × 100 mm, Waters, Milford, MA, USA). The equipment was controlled through the software application EmpowerTM 3 (Waters Corporation, Milford, MA, USA).

In order to determine and quantify the phenolic compounds in the extract, 3.0 μL samples were injected into the column, and the eluent was allowed to flow at 0.6 mL min^−1^. The eluent consisted of a mixture of Milli-Q water and HPLC-grade acetonitrile, both acidified using 2% glacial acetic acid. The gradient used has already been described by Yerena-Prieto et al. [36]: 0 min, 0% B; 1 min, 5% B; 2 min, 10% B; 3 min, 15% B; 4 min, 20% B; 5 min, 30% B; 7 min, 35% B; 8 min, 40% B; 10 min, 75% B and 12 min, 0% B. The total analysis time, including the return to the initial conditions and re-equilibration, spanned 15.0 min.

The compounds were quantified at *λ* = 350 nm, based on a calibration curve (*y* = 6007.6*x* + 1930.3; *R*^2^ = 0.9992) generated by 0.5, 1, 5, 10 and 50 mg L^−1^ Q3GLU in methanol, as this is one of the main phenolic compounds produced by *M. oleifera* [30]. The results were therefore expressed as milligrams of Q3GLU equivalents per gram of dried leaves (mg Q3GLUE/g).

### 2.5. Extraction Method

#### 2.5.1. Placket–Burman Experimental Design

The Placket–Burman Experimental Design is a screening method that allows researchers to reduce the number of factors required for an experimental design by pointing out which of the factors has a significant influence. Thus, based on a statistical algorithm, the number of experiments required to identify which relevant factors can be reduced [37]. In this particular case, instead of the 128 (2^7^) experiments that would be required for a factorial design determined by 7 factors and 2 levels (−1.0, lower and 1.0 higher), just 12 experiments were needed to determine the factors to be taken into account for an optimal procedure (Table 1).

As already mentioned in Section 2.3.2, the factors selected for this study were time (*X*_1_, expressed as min), pH (*X*_2_), temperature (*X*_3_, expressed as °C), agitation (*X*_4_, expressed as revolutions per minute) solvent composition (*X*_5_, expressed as ethanol percentage), sample-to-solvent ratio (*X*_6_, expressed as grams sample per 15 mL solvent) and enzyme-to-sample ratio (*X*_7_, expressed as units of enzyme per gram sample). The response variable (*Y*) for the design of the method was the sum of the peak areas, given that the ultra-high-performance liquid chromatography-photodiode array detector (UHPLC-PDA) method is more reliable than other colorimetric quantification techniques.

#### 2.5.2. Box–Behnken Experimental Design

Once the most significant factors had been identified, it was necessary to determine the optimal value for each factor. A Box–Behnken Design determined by 4 factors (solvent pH (*X*_2_), temperature (*X*_3_), solvent composition (*X*_5_) and enzyme-to-sample ratio (*X*_7_)) at 3 levels (−1.0, low; 0, medium and 1.0, high) (Table 2) was employed for that purpose. As already mentioned in Section 2.5.1., the sum of the peak areas related to the mass of dry sample employed was considered as the response variable (*Y*) to be optimized for the design of the extraction method.

This statistical design allows reducing the number of experiments to 27 compared to the 81 (3^4^) experiments that would have been required for an equivalent factorial design. Thus, the maximum information is obtained from a relatively shorter number of experiments. This is a spherical design, where the distance from the experimental points to the central one is α = √2, while no experiments under extreme conditions are required, which is rather convenient, since those conditions that might threaten the integrity of some of the compounds in the samples, such as high temperatures, are left out [37].

(1) $$Y=\beta_{0}+\overset{k}{\underset{i=1}{\sum }}(\beta_{i}X_{i}+\beta_{ii}X_{i}{}^{2})+\overset{n}{\underset{i<j}{\sum }}\overset{k}{\underset{i=1}{\sum }}\beta_{ij}X_{i}X_{j}+r$$

The experimental results were adjusted to a surface-response polynomium (Equation (1)) where 𝛽_𝑖_ is the coefficient assigned to the main effects; 𝛽_𝑖𝑗_, to the interaction effects; 𝛽_𝑖𝑖_, to the quadratic factors, *X_i_* and *X_j_* correspond to each factor, and *r* is the residual value. The fit of the model is indicated by the *R*^2^ coefficient obtained, and the statistical significance of each factor can be determined through an analysis of variance (ANOVA). The data obtained from all the experiments were analyzed by means of the statistical software application STATGRAPHICS XVI (Statgraphics Technologies, Inc., The Plains, VA, USA).

#### 2.5.3. Determining the Extraction Time

After establishing the optimal extraction conditions, a single-factor study was performed in order to determine the optimal extraction time. For that purpose, a number of extractions under the established optimal conditions were performed in triplicate using 2, 5, 10, 15, 20 and 25 min.

#### 2.5.4. Repeatability and Intermediate Precision

Three extraction batches of 8 replicates each were performed under the established optimal conditions in order to evaluate the repeatability and the intermediate precision of the developed method. Each batch was subjected to extraction on a different day so that the relative standard deviation (RSD) corresponding to the intragroup repeatability (within each batch) and the RSD corresponding to the intergroup intermediate precision could be determined.

### 2.6. Determining the Antioxidant Compounds Content

The flavonoids content in the final extracts that had been obtained under the established optimal conditions was measured in order to assess the antioxidant properties of the extracts and the suitability of the extraction method for the intended purpose.

#### 2.6.1. Quantifying the Antioxidant Compounds through Ultra-High Performance Liquid Chromatography (UHPLC)

**Figure 1:**
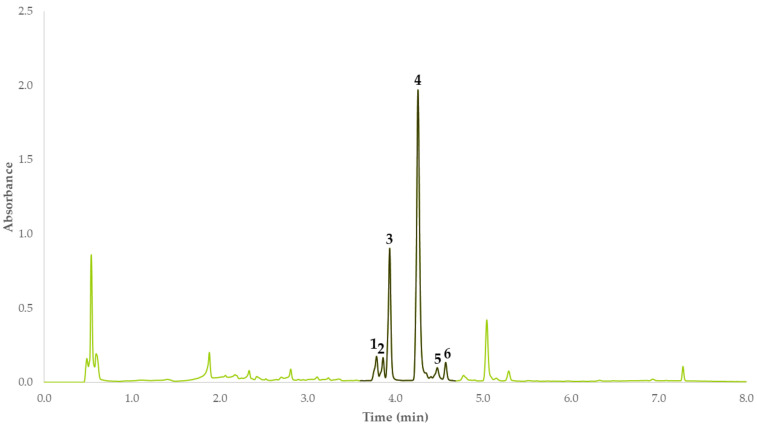
Chromatogram of a *M. oleifera* extract at *λ* = 350 nm. Q3GLU (**1**): quercetin 3-*O*-glucoside; QMGLU; (**2**): quercetin malonyl glucoside; QHMGGLU (**3**): quercetin hydroxy methyl glutaroyl glucoside; QAGLU (**4**): quercetin acetyl glucoside; K3GLU (**5**): kaempferol-3-glucoside; I3GLU (**6**): isorhamnetin-3-glucoside.

The flavonoids were identified through their chromatograms at 350 nm (Figure 1). Only the compounds that could be detected at a retention time between 3.60 and 4.50 min were considered, as this is the region where these compounds are to be found when this chromatographic method is used [30].

This chromatographic method was used to identify the compounds based on their retention times and according to previous studies completed by our research group [30], and the results were expressed as milligrams of Q3GLU per gram sample, as this is the main phenolic compound produced by moringa.

#### 2.6.2. Evaluation of the Antioxidant Potential of the Extracts through Colorimetric Methods

In addition to the UHPLC-PDA quantification, the antioxidant activity of the final extracts was determined using two different colorimetric methods: free radical scavenging activity through a 2,2-diphenyl-1-pycrylhydrazile (DPPH) assay and total phenolic content through a Folin–Ciocalteu assay.

##### Determining the Free Radical Scavenging Activity

This assay was performed as follows: a 100 μL sample was gently mixed with 2 mL of a 6 × 10^−5^ M stock solution of DPPH. The reaction was left in the absence of light for 40 min, and then its absorbance at *λ* = 515 nm was measured by means of a UV-Vis Helios-γ-Unicam spectrophotometer (Thermo Scientific, Waltham, MA, USA) in 10 mm-width cuvettes. A blank solution was prepared by replacing the sample with distilled water. The free radical scavenging activity was determined according to Equation (2), where *C* is the free radical scavenging activity; *A*_0_, the blank absorbance and *A*_S_ the absorbance of the sample, both absorbances measured at 515 nm.

(2) $$C=\frac{A_{0}-A_{S}}{A_{0}}\times 100\%,$$

Once the free radical scavenging activity had been determined, the concentration of the antioxidant compounds could be measured based on a calibration curve (*y* = 88.941*x* + 0.7478; *R*^2^ = 0.9959) obtained by measuring the free radical scavenging activity of the Trolox standard solutions at 0.0, 0.3, 0.6, 0.9 and 1.1 mM. Trolox is widely used as a standard for antioxidant-determining assays [38,39,40,41], as it is the hydrosoluble analogue of vitamin E [42]. Consequently, the results were expressed in terms of milligrams of Trolox equivalents per gram sample (mg TE/g).

##### Determining Total Phenolic Content (TPC)

The TPC can be determined by attending to the interpolation of the absorbance data of the calibration curve (*y* = 0.0015*x* + 0.0059; *R*^2^ = 0.9999) previously generated and by measuring the TPC of a number of gallic acid solutions at 25, 50, 100, 250, 500 and 1000 mg L^−1^, since gallic acid is a phenolic compound typically employed as the reference for these assays [43,44,45]. The results are then expressed as milligrams of gallic acid equivalents per gram sample (mg GAE/g).

## 3. Results and Discussion

### 3.1. Developing an Optimized Extraction Method for Moringa Antioxidant Compounds

#### 3.1.1. Placket–Burman Experimental Design

Seven factors were considered for the development and optimization of the EE method: time (*X*_1_, expressed as min), pH (*X*_2_), temperature (*X*_3_, expressed as °C), agitation (*X*_4_, expressed as revolutions per minute) solvent composition (*X*_5_, expressed as ethanol percentage), sample-to-solvent ratio (*X*_6_, expressed as grams sample per 15 mL solvent) and enzyme-to-sample ratio (*X*_7_, expressed as enzyme units per gram sample).

In order to determine the influence from each factor on the flavonoids extraction yields, the abovementioned factors were screened considering two levels for each factor. This design requires a total of 12 extractions to be carried out. The response variable was expressed as standardized areas (area units per gram sample; AU/g) where the chromatographic peaks comprising between 3.60 and 4.50 min were considered, so that the influence from each factor on the final yield could be determined.

**Figure 2:**
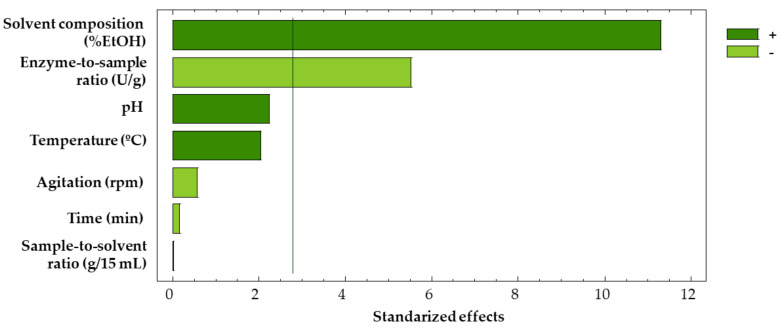
Pareto diagram of the Placket–Burman screening design.

The experimental data were subjected to ANOVA (Table 3) in order to determine the influence from each factor on the extraction yield. It was revealed from the resulting Pareto diagram (Figure 2) that only the solvent composition and the enzyme-to-sample ratio were significant factors. Nevertheless, pH and temperature were also considered for the Box–Behnken design, as Placket–Burman designs may provide a biased picture given that only the extreme values are considered, while the effect of the mild value factors are disregarded.

**Figure 3:**
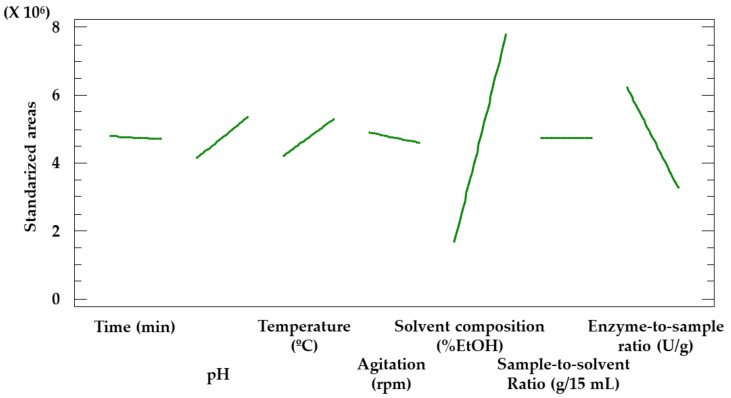
Plot representing the main effects of the 7 factors screened by the Placket–Burman design.

The plot corresponding to the most significant effect (Figure 3) allows us to determine the value that should be assigned to each factor for further analysis. As the extraction yields were maximized when ethanol-rich solvents were used, the range of values to be studied was adjusted from 0−40% to 20−60% in order to determine if higher ethanol percentages would lead to greater extraction yields. With regard to the rest of the factors, the values to be considered remained unchanged.

Given that agitation, sample-to-solvent ratio or time did not prove to be significant factors, the values to be established for further analysis were determined according to whether their effect on the extraction yield was positive (sample-to-solvent ratio) or negative (agitation and time). Therefore, 50 rpm, 0.2 g/15 mL and 10 min were established as the values to be used for the Box–Behnken design extractions.

*Y* = −2.00295 × 10^6^ − 1.85821 × 10^4^ × *X*_1_ + 6.11782 × 10^5^ × *X*_2_ + 5.58624 × 10^4^ × *X*_3_ − 2.08245 × 10^3^ × *X*_4_ + 1.53801 × 10^5^ × *X*_5_ − 8.39442 × 10^4^ × *X*_6_ − 3.33783 × 10^3^ × *X*_7_ (3)

Based on the statistical analysis, a polynomial (Equation (3)), where 97.67% was the *R*^2^ coefficient, was also obtained. This high *R*^2^ value confirms the narrow difference between the experimental and the predicted values and, therefore, the suitability of the model to predict the standardized areas that would result from each specific combination of factor values.

#### 3.1.2. Box–Behnken Experimental Design

**Figure 4:**
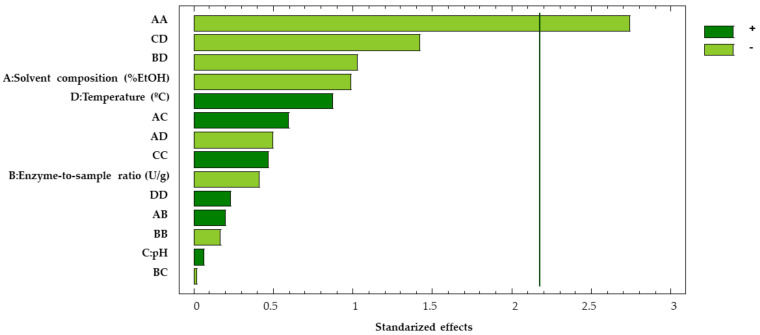
Pareto chart of the Box–Behnken surface-response design.

The data obtained from the Box–Behnken design were subjected to ANOVA (Table 4). It can be observed from the Pareto chart in Figure 4 that only the quadratic effect of the solvent composition was to be considered as significant, while the rest of the factors produced values that remained in every case below the significant threshold (*p*-values > 0.05) (2.78). Therefore, it can be assumed that solvent is a relevant factor for the extraction procedure despite not being a significant factor itself. This result was to be expected, as the composition of the extraction solvent is one of the most influential parameters when extracting biological compounds in plant matrices. In this sense, solvents or mixtures of solvents with a similar polarity to the compounds to be extracted should be used.

**Figure 5:**
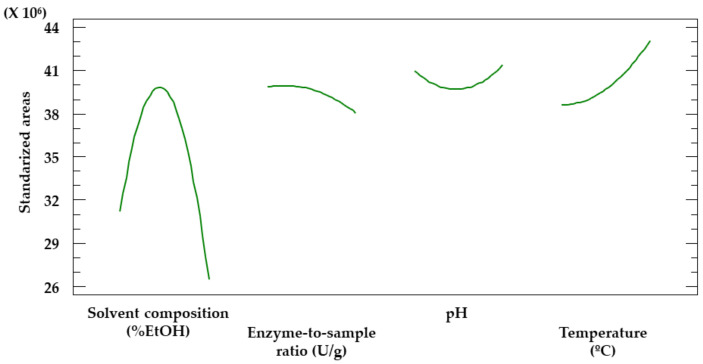
Plot representing the main effects of solvent composition, enzyme-to-sample ratio, pH and temperature.

Based on the plot of the main effects in the Box–Behnken design (Figure 5), the optimal value for each factor can be determined. These values are displayed in Table 5.

As mentioned above, the values for the agitation and the sample-to-solvent ratio were determined based on the plot corresponding to the main effects in the Placket–Burman design (Figure 3). Thus, the optimal pH (4.0) matched the minimal value considered in this study. Given that pectinases extraction yields decrease when pH is above 5.0 [46] and, as stated in the technical sheet of the product, its optimal pH is near 4.0, no further assays were needed to determine the optimal pH level for the extractions. The optimal temperature (58.9 °C) is very close to the maximum value studied (60 °C), as phenolic compounds are thermostable even at high temperatures [47,48] in this short period of time. The optimal solvent composition (32.5% EtOH) was at an intermediate value, as the solvent determines the conformation the enzyme acquires in its matrix. Therefore, it conditions the activity of the enzyme [49]. Finally, the optimal enzyme-to-sample ratio (106 U/g) is near the minimum value studied (100 U/g), as could also be guessed from the Placket–Burman Pareto (Figure 2), where the enzyme-to-sample ratio showed a negative effect on the extraction yield. Greater ratios may lead to enzymatic saturation [50].

*Y* = −1.66992 × 10^8^ + 1.80908 × 10^6^ × *X*_5_ + 1.46718 × 10^5^ × *X*_7_ + 7.95647 × 10^6^ × *X*_2_ + 4.28385 × 10^6^ × *X*_3_ − 2.69988 × 10^4^ × *X*_5_^2^ + 9.43124 × 10 × *X*_5_ × *X*_7_ + 1.88108 × 10^5^ × *X*_2_ × *X*_5_ − 1.51726 × 10^4^ × *X*_3_ × *X*_5_ − 3.57992 × *X*_7_^2^ − 2.03787 × 10^2^ × *X*_2_ × *X*_7_ − 2.95035 × 10^3^ × *X*_3_ × *X*_7_ + 1.47627 × 10^6^ × *X*_2_^2^− 5.98717 × 10^5^ × *X* × *X*_3_ + 1.16086 × 10^4^ × *X*_3_^2^ (4)

The *R*^2^ coefficient of the polynomial (Equation (4)) obtained was just 68.56%, which means that the predictability of the model is not high enough to predict the standardized areas based on a specific combination of values assigned to each influent factor.

#### 3.1.3. Determining the Extraction Time

**Figure 6:**
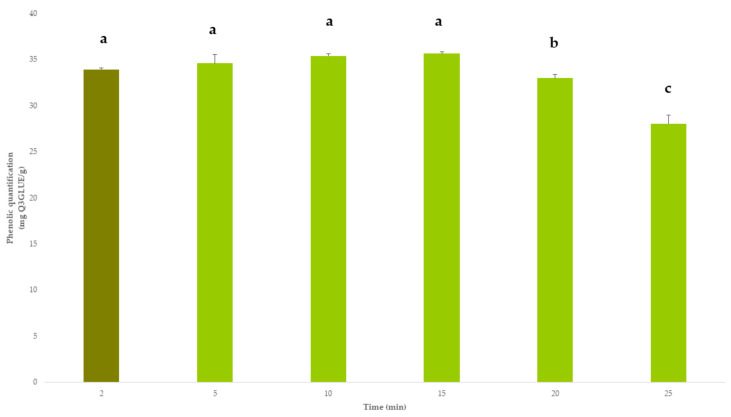
Single-factor study to determine the optimal extraction time. Different letters “a–c” indicate significant differences between results at the 95% confidence level according to Tukey’s test.

Once the method had been optimized, a single-factor experiment was performed to determine the optimal extraction time. The extractions were performed in triplicate using 2, 5, 10, 15, 20 and 25 min. The results are displayed in Figure 6.

An analysis of variance (ANOVA) was applied to the data in order to identify any possible relevant differences between the yields obtained using different times. Since no significant differences were detected (*p*-values > 0.05), 2 min was established as the best extraction time because of its lower energy and resources demand.

#### 3.1.4. Repeatability and Intermediate Precision

Once all of the optimal conditions had been established, the repeatability (*n* = 8) and the intermediate precision (*n* = 8 + 8 + 8) of the extraction method were evaluated and established at 1.39% and 2.37%, respectively. As these percentages were below 5%, the extraction method was considered as suitable in terms of repeatability and precision [51].

### 3.2. Determining the Antioxidant Compounds

As already mentioned in Section 2.6., the antioxidant compounds in the extracts obtained under optimal conditions (Table 6) were determined by chromatographic (UHPLC-PDA) and colorimetric (DPPH and Folin–Ciocalteu) methods.

It can be observed from the data displayed in Table 6 that both measuring methods provided positive data. It can therefore be considered that the extracts obtained hold antioxidant properties and that the EE method is adequate for the production of extracts with antioxidant properties. Furthermore, the optimal extraction conditions that had been established did not threaten the antioxidant capacities of the extracts, as the integrity of the phenolic compounds had been preserved.

The method herein developed results in an innovative approach for the obtention of antioxidant flavonoids from moringa samples. It is a green and fast method as it implies low energetic consumption as it uses mild temperatures (58.9 °C) and low amounts of organic solvent per sample (4.875 mL ethanol per processed sample), and it only consumes approximately 15 min per sample.

## 4. Conclusions

An EE method with good repeatability and intermediate precision (RSD < 5%) has been developed in this study that allows moringa extracts with antioxidant properties to be obtained (23.83 ± 3.10 mg Q3GLUE/g of sample; 4.664 ± 0.003 mg TE/g of sample and 6.245 ± 0.002 mg GAE/g of sample) using mild temperatures (58.9 °C) and short extraction times (2 min). These mild values allow obtaining these bioactive extracts while considerably saving costs and resources.

Furthermore, these extracts can be used as a starting point for the identification of lead compounds for the development of drugs to treat illnesses related to oxidative stress such as diabetes and cardiovascular diseases.

## Figures and Tables

**Table 1:** Experimental and predicted values of the standardized areas based on a Placket–Burman screening design of the enzymatic extraction.

Run	Factors							Response (Sum of Peak Areas)	
	*X*_1_	*X*_2_	*X*_3_	*X*_4_	*X*_5_	*X*_6_	*X*_7_	Experimental	Predicted
1	1.0	−1.0	1.0	1.0	−1.0	1.0	−1.0	1.03095 × 10^6^	1.2298 × 10^6^
2	−1.0	1.0	−1.0	−1.0	−1.0	1.0	1.0	1.07526 × 10^7^	1.06054 × 10^7^
3	1.0	−1.0	1.0	−1.0	−1.0	−1.0	1.0	5.6065 × 10^6^	6.16669 × 10^6^
4	1.0	1.0	1.0	−1.0	1.0	1.0	−1.0	7.06965 × 10^6^	6.1801 × 10^6^
5	−1.0	1.0	1.0	−1.0	1.0	−1.0	−1.0	8.62203 × 10^6^	8.04509 × 10^6^
6	−1.0	−1.0	−1.0	−1.0	−1.0	−1.0	−1.0	8.11025 × 10^5^	6.34091 × 10^5^
7	−1.0	1.0	1.0	1.0	−1.0	1.0	1.0	6.23779 × 10^5^	4.24924 × 10^5^
8	−1.0	−1.0	−1.0	1.0	1.0	1.0	−1.0	3.09949 × 10^6^	2.91737 × 10^6^
9	−1.0	−1.0	1.0	1.0	1.0	−1.0	1.0	1.05594 × 10^7^	1.07067 × 10^7^
10	1.0	1.0	−1.0	1.0	1.0	−1.0	1.0	2.3443 × 10^6^	2.2138 × 10^6^
11	1.0	1.0	−1.0	1.0	−1.0	−1.0	−1.0	2.14253 × 10^6^	3.03208 × 10^6^
12	1.0	−1.0	−1.0	−1.0	1.0	1.0	1.0	4.35421 × 10^6^	5.26051 × 10^6^

**Table 2:** Actual and predicted values of the standardized areas based on the Box–Behnken surface-response design of the enzymatic extraction.

Run	Factors				Response (Sum of Peak Areas)	
	*X*_2_	*X*_3_	*X*_5_	*X*_7_	Experimental	Predicted
1	0.0	0.0	0.0	0.0	4.12729 × 10^7^	3.8611 × 10^7^
2	−1.0	0.0	1.0	0.0	4.46509 × 10^7^	3.03722 × 10^7^
3	−1.0	0.0	−1.0	0.0	4.09369 × 10^7^	3.62496 × 10^7^
4	−1.0	0.0	0.0	1.0	3.77866 × 10^7^	3.94202 × 10^7^
5	0.0	0.0	1.0	−1.0	2.10849 × 10^7^	2.58438 × 10^7^
6	1.0	0.0	−1.0	0.0	2.53149 × 10^7^	3.10147 × 10^7^
7	0.0	1.0	0.0	1.0	3.67347 × 10^7^	3.80478 × 10^7^
8	0.0	0.0	1.0	1.0	2.33049 × 10^7^	2.57562 × 10^7^
9	0.0	0.0	0.0	0.0	4.35064 × 10^7^	3.96654 × 10^7^
10	0.0	0.0	−1.0	−1.0	3.13069 × 10^7^	3.22233 × 10^7^
11	1.0	0.0	1.0	0.0	4.38178 × 10^7^	3.99262 × 10^7^
12	0.0	1.0	−1.0	0.0	2.48077 × 10^7^	2.71178 × 10^7^
13	1.0	1.0	0.0	0.0	3.75622 × 10^7^	3.87262 × 10^7^
14	−1.0	0.0	0.0	−1.0	1.93126 × 10^7^	2.07039 × 10^7^
15	−1.0	1.0	0.0	0.0	3.7854 × 10^7^	4.09461 × 10^7^
16	1.0	0.0	0.0	−1.0	4.21155 × 10^7^	4.15966 × 10^7^
17	0.0	−1.0	−1.0	0.0	2.99655 × 10^7^	2.71178 × 10^7^
18	0.0	−1.0	1.0	0.0	2.30329 × 10^7^	2.68665 × 10^7^
19	0.0	1.0	1.0	0.0	1.93985 × 10^7^	2.65249 × 10^7^
20	0.0	0.0	−1.0	1.0	2.32658 × 10^7^	2.18745 × 10^7^
21	1.0	−1.0	0.0	0.0	4.09029 × 10^7^	3.87262 × 10^7^
22	1.0	0.0	0.0	1.0	3.99046 × 10^7^	3.9628 × 10^7^
23	0.0	−1.0	0.0	1.0	4.17779 × 10^7^	3.80478 × 10^7^
24	−1.0	−1.0	0.0	0.0	3.74603 × 10^7^	5.03094 × 10^7^
25	0.0	0.0	0.0	0.0	4.13555 × 10^7^	3.96654 × 10^7^
26	0.0	1.0	0.0	−1.0	4.23928 × 10^7^	3.9833 × 10^7^
27	0.0	−1.0	0.0	−1.0	4.38209 × 10^7^	3.9833 × 10^7^

**Table 3:** Analysis of variance (ANOVA) of the Placket–Burman screening design.

Factor	Factor Code	Coefficients	Sum of Squares (10^11^)	Degrees of Freedom	Mean Square (10^10^)	*F*-Value	*p*-Value
Model		−2.00295 × 10^6^					
A: Time	*X*_1_	−1.85821 × 10^4^	2.58972 × 10^−1^	1	2.58972	0.03	0.8728
B: Ph	*X*_2_	6.11782 × 10^5^	4.49133 × 10	1	4.49133 × 10^2^	5.05	0.0879
C: Temperature	*X*_3_	5.58624 × 10^4^	3.74473 × 10	1	3.74473 × 10^2^	4.21	0.1094
D: Agitation	*X*_4_	−2.08245 × 10^3^	2.92722	1	2.92722 × 10	0.33	0.5968
E: Solvent composition	*X*_5_	1.53801 × 10^5^	1.13544 × 10^3^	1	1.13544 × 10^4^	127.71	0.0003
F: Sample-to-solvent ratio	*X*_6_	−8.39442 × 10^4^	2.11399 × 10^−3^	1	2.11399 × 10^−2^	0.00	0.9884
G: Enzyme-to-sample ratio	*X*_7_	−3.33783 × 10^3^	2.70728 × 10^2^	1	2.70728 × 10^3^	30.45	0.0053
Total error			3.55616 × 10	4	8.8904 × 10		
Total correlation			1.52727 × 10^3^	11			

**Table 4:** ANOVA of the Box–Behnken surface-response design.

Factor	Factor Code	Coefficients	Sum of Squares (10^11^)	Degrees of Freedom	Mean Square (10^10^)	*F−*Value	*p−*Value
Model		−1.66992 × 10^8^					
A: Solvent composition	*X*_5_	1.80908 × 10^6^	5.09363 × 10^2^	1	5.09363 × 10^3^	7.29	0.0428
B: Enzyme-to-sample ratio	*X*_7_	1.46718 × 10^5^	8.86773 × 10	1	8.86773 × 10^2^	1.27	0.3110
C: pH	*X*_2_	7.95647 × 10^6^	2.33719	1	2.33719 × 10	0.03	0.8621
D: Temperature	*X*_3_	4.28385 × 10^6^	4.02734 × 10^2^	1	4.02734 × 10^3^	5.76	0.0615
AA	*X*_5_^2^	−2.69988 × 10^4^	3.95557 × 10^3^	1	3.95557 × 10^4^	56.62	0.0007
AB	*X*_5_ *X*_7_	9.43124 × 10	2.14369 × 10	1	2.14369 × 10^2^	0.31	0.6035
AC	*X*_2_ *X*_5_	1.88108 × 10^5^	1.8915 × 10^2^	1	1.8915 × 10^3^	2.71	0.1608
AD	*X*_3_ *X*_5_	1.51726 × 10^4^	1.28104 × 10^2^	1	1.28104 × 10^3^	1.83	0.2337
BB	*X*_7_^2^	−3.57992	1.44392 × 10	1	1.44392 × 10^2^	0.21	0.6684
BC	*X*_2_ *X*_7_	−2.03787 × 10^2^	2.44845 × 10^−1^	1	2.44845	0.00	0.9551
BD	*X*_3_ *X*_7_	−2.95035 × 10^3^	5.54794 × 10^2^	1	5.54794 × 10^3^	7.94	0.0372
CC	*X*_2_^2^	1.47627 × 10^6^	1.1477 × 10^2^	1	1.1477 × 10^3^	1.64	0.2562
CD	*X*_2_ *X*_3_	−5.98717 × 10^5^	1.06067 × 10^3^	1	1.06067 × 10^4^	15.18	0.0115
DD	*X*_3_^2^	1.16086 × 10^4^	2.85304 × 10	1	2.85304 × 10^2^	0.41	0.5509
Lack of fit			5.97405 × 10^3^	7	8.53435 × 10^3^	12.22	0.0070
Pure error			3.49311 × 10^2^	5	6.98622 × 10^2^		
Total correlation			2.01122 × 10^4^	26			

**Table 5:** Optimal values of the relevant variables in enzymatic extractions.

Factor	Optimal Value
pH	4.0
Temperature (°C)	58.9
Agitation (rpm)	50
Solvent composition (%EtOH)	32.5
Sample-to-solvent ratio (g/15 mL)	0.2
Enzyme-to-sample ratio (U/g)	106

**Table 6:** Quantification of the antioxidant compounds in the *M. oleifera* extracts and determination of their antioxidant potential.

Method	Unit	Obtained Value
UHPLC-PDA	mg Q3GLUE/g	23.83 ± 3.10
DPPH	mg TE/g	4.664 ± 0.059
Folin–Ciocalteu	mg GAE/g	6.245 ± 0.101

## Data Availability

The data presented in this study are contained within the article.
